# Loss-of-function N178T variant of the human P2Y_4_ receptor is associated with decreased severity of coronary artery disease and improved glucose homeostasis

**DOI:** 10.3389/fphar.2022.1049696

**Published:** 2022-12-02

**Authors:** Michael Horckmans, Esteban Diaz Villamil, Céline Verdier, Henrik Laurell, Jean-Bernard Ruidavets, Lucas De Roeck, Guillaume Combes, Laurent O. Martinez, Didier Communi

**Affiliations:** ^1^ Institute of Interdisciplinary Research, IRIBHM, Free University of Brussels, Brussels, Belgium; ^2^ Institut des Maladies Métaboliques et Cardiovasculaires, I2MC, Inserm, Université de Toulouse, Université Toulouse III—Paul Sabatier (UPS), UMR1297, Toulouse, France; ^3^ CHU de Toulouse, Toulouse University Hospital, Toulouse, France; ^4^ CERPOP UMR, Inserm, University of Toulouse III, UPS, Toulouse, France

**Keywords:** P2Y receptor, cardioprotection, extracellular nucleotides, glucose homeostasis, cardiac ischemia

## Abstract

Human P2Y_4_ is a UTP receptor, while in mice it is activated by both ATP and UTP. P2Y_4_ knockout (KO) in mice protects against myocardial infarction and is characterized by increased adiponectin secretion by adipocytes, and decreased cardiac inflammation and permeability under ischemic conditions. The relevance of these data has, however, not been explored to date in humans. In a population study comprising 50 patients with coronary artery disease (CAD) and 50 age-matched control individuals, we analyzed *P2RY4* mutations and their potential association with CAD severity and fasting plasma parameters. Among the mutations identified, we focused our attention on a coding region polymorphism (rs3745601) that results in replacement of the asparagine at residue 178 with threonine (N178T) located in the second extracellular loop of the P2Y_4_ receptor. The N178T variant is a loss-of-function mutation of the human P2Y_4_ receptor and is encountered less frequently in coronary patients than in control individuals. In coronary patients, carriers of the N178T variant had significantly reduced jeopardy and Gensini cardiac severity scores, as well as lower resting heart rates and plasma levels of N-terminal pro-brain natriuretic peptide (NT-proBNP). Regarding fasting plasma parameters, the N178T variant was associated with a lower concentration of glucose. Accordingly, P2Y_4_ KO mice had significantly improved glucose tolerance and insulin sensitivity compared with their WT littermate controls. The improvement of insulin sensitivity resulting from lack of the P2Y_4_ receptor was no longer observed in the absence of adiponectin. The present study identifies a frequent loss-of-function P2Y_4_ variant associated with less severe coronary artery atherosclerosis and lower fasting plasma glucose in coronary patients. The role of the P2Y_4_ receptor in glucose homeostasis was confirmed in mouse. P2Y_4_ antagonists could thus have therapeutic applications in the treatment of myocardial infarction and type 2 diabetes.

## Introduction

Patients suffering from coronary artery disease represent a heterogeneous group, with major differences in comorbidities and other metabolic factors such as insulin resistance. The risks of individual patients and the potential benefit they will experience from specific treatments depend on these various factors. The characterization of specific gene mutations in patients with adverse outcomes could improve the identification of patients at higher risk, as well as the follow-up after myocardial infarction (MI).

The human P2Y_4_ subtype is a UTP receptor, originally cloned in our laboratory (Communi et al., 1995), and is activated by both ATP and UTP in mouse (Suarez-Huerta et al., 2001). We previously observed that the heart of mice deficient for the P2Y_4_ subtype receptor had a significantly reduced size at adult age due to an angiogenic defect in the early days of postnatal development (Horckmans et al., 2012a). We also showed that P2Y_4_ KO mice have decreased resistance in forced exercise tests with cardiac monitoring (Horckmans et al., 2012b). In another study, we observed that P2Y_4_ KO mice are protected against myocardial infarction: they display smaller infarcts in the left anterior descending coronary artery ligation model (LAD ligation model), as well as a significant decrease in cardiac inflammation and permeability (Horckmans et al., 2015). Interestingly a higher level of adiponectin, which is a cardioprotective adipokine, correlated with an increased mass of cardiac adipose tissue in P2Y_4_ KO mice (Horckmans et al., 2015; Lemaire et al., 2017).

Several studies have been undertaken to assess the genetic risk for MI in individuals independently of conventional risk factors (Nishihama et al., 2007). Different polymorphisms related to MI risk have been identified in genes such as glycoprotein Ib alpha, insulin promoter factor 1, and methylenetetrahydrofolate reductase, as well as in many other genes (Nishihama et al., 2007; Sakowicz et al., 2013). Other studies have focused on the association of polymorphisms in a particular gene, such as angiotensinogen or angiotensin-converting enzyme, and MI risk (Hamelin et al., 2011; Li et al., 2021). More specifically, *AGT* p.Thr174Met may increase the risk for MI, especially in the Asian population (Li et al., 2021), and the ACE-I/D polymorphism appears to be a genetic risk factor for MI at a young age (Hamelin et al., 2011). Several mutations have already been identified in P2Y receptor subtypes such as the P2Y_12_ and P2Y_1_ receptors (Lev et al., 2007), the P2Y_11_ receptor (Amisten et al., 2007), and the P2Y_13_ receptor (Amisten et al., 2008; Verdier et al., 2019). Only the Ala-87-Thr variant (rs3732757) of the human ATP receptor P2Y_11_ has been associated with an increased risk of acute MI (Amisten et al., 2007), while the synonymous variant Ile-80-Ile (rs3732757) of the human ADP receptor P2Y_13_ is associated with increased fat mass and a lower heart rate (Verdier et al., 2019).

Besides well-established leading risk factors for heart disease such as diabetes, obesity, high blood pressure and high level of low-density lipoprotein cholesterol (LDL-C), N-terminal pro-brain natriuretic peptide (NT-proBNP), a cardiac marker indicative of myocardial damages, has been related to heart failure severity and insulin resistance (Palazzuoli et al., 2010; Baldassarre et al., 2017). The present study aimed to analyze association of a common P2Y_4_ receptor missense mutation with markers of cardiometabolic health and with the severity of CAD.

## Materials and methods

### Study sample

The GENES (Génétique et ENvironnement en Europe du Sud) study is a case–control study designed to assess the role of genetic, biological, and environmental determinants in the occurrence of CAD. As previously described (Genoux et al., 2016), all participants were men, recruited from 2001 to 2004, aged 45–74 years and living in the Toulouse area (south-western France). Stable CAD patients (cases) were recruited after admission to the Department of Cardiology of Toulouse University Hospital and control subjects were selected from the general population using electoral rolls. Stratification into decadal age groups was used to match the age distribution of the people with and without stable CAD. Stable CAD was defined as a history of acute coronary syndrome, a history of coronary artery revascularization, documented myocardial ischemia, stable angina, or the presence upon coronary angiography of coronary stenosis of 50% or more. Diffusion of coronary heart disease lesions was assessed by calculation of the Gensini score and the Duke jeopardy score based on data from coronary angiography (Califf et al., 1985). All participants underwent a medical examination at the same health center during the same period, including clinical and anthropometric measurements. Resting heart rate was measured after ≥5 min of rest, using an automatic sphygmomanometer and systolic blood pressure was detected with a hand-held Doppler probe. Information on cardiovascular risk factors were collected through standardized face-to-face interviews, performed by a single physician. Presence of dyslipidemia, diabetes mellitus or hypertension was assessed from the subjects’ current treatments. Past medical history was collected and, for cases, was also checked in the patients’ medical files. Patients’ medications at discharge were also considered. Blood was collected after an overnight fast, and a blood sample collection was constituted. Fasting plasma lipids, glucose, N-terminal pro-brain natriuretic peptide (NT-proBNP) and high-sensitivity cardiac troponin T (hs-TnT) were assayed with enzymatic reagents on automated analyzers (Hitachi 912 and Cobas 8000^®^; Roche Diagnostics, Meylan, France) (Genoux et al., 2016).

In the present study, the *P2RY4* gene was sequenced in 50 randomly selected CAD patients from the initial sample, age-matched to 50 control subjects (mean age 61.17 ± 7.26 and 60.55 ± 8.14 years, respectively).

### *P2RY4* sequencing and mutation analyses

**FIGURE 1:**
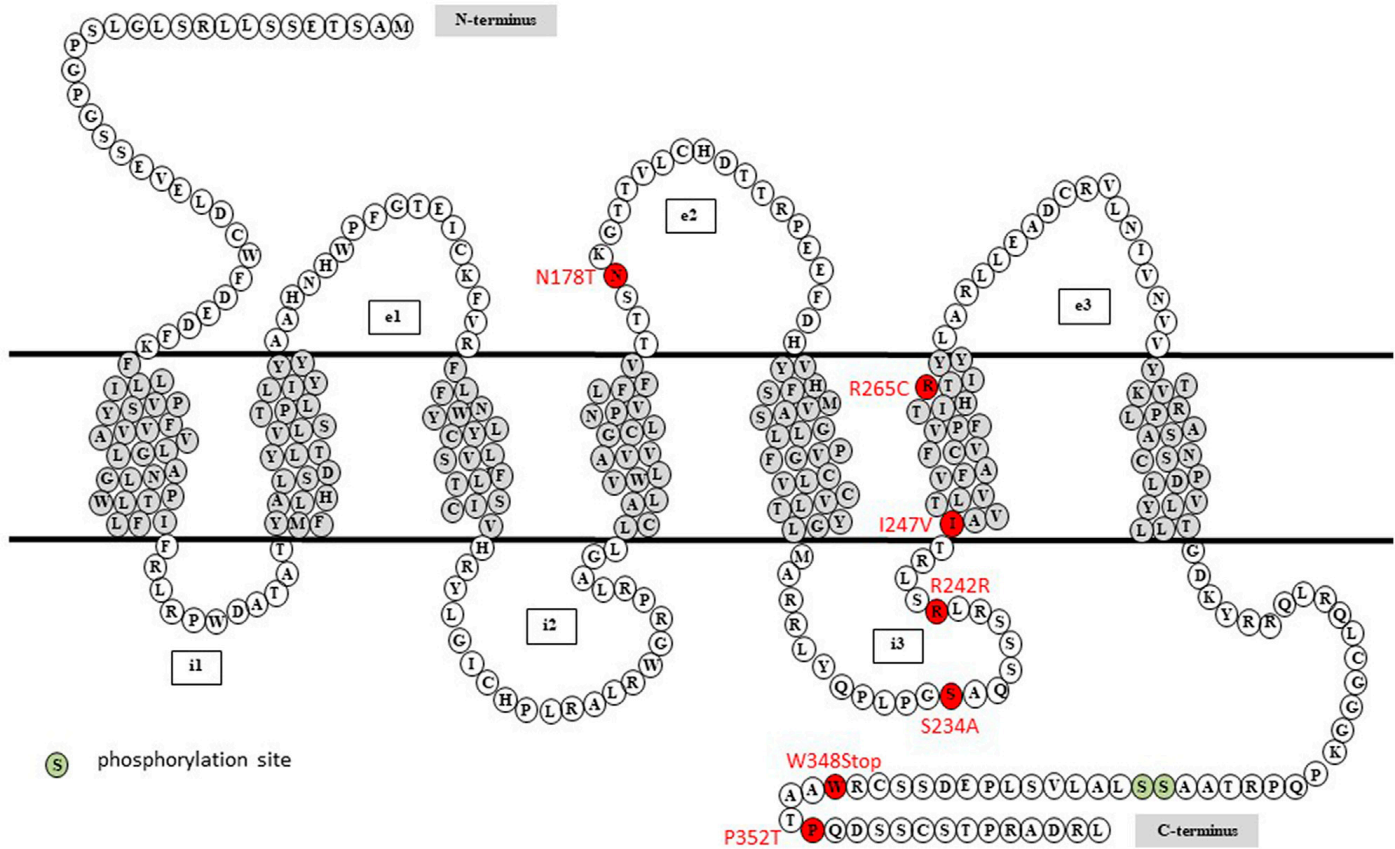
Localization of the seven mutations corresponding to identified polymorphisms in the human *P2RY4* gene in individuals included in the GENES study. Among the identified variants (in red), the N178T polymorphism is located in the second extracellular loop (e2), S234A and R242R in the third intracellular loop (i3), I247V and R265C in the sixth transmembrane domain, and W348Stop and P352T in the C-terminus of the human P2Y_4_ receptor amino acid sequence. Potential phosphorylation sites are indicated in green.

Genomic deoxyribonucleic acid (gDNA) was isolated from ethylenediaminetetraacetic acid (EDTA)-treated blood samples using silica columns (NucleoSpin^®^ Extract II; Macherey-Nagel, Duren, Germany). *P2RY4* is a 2.04 kb gene located in the q13 region of the X chromosome and is composed of one exon encoding a 365-amino acid protein (Supplementary Material; Figure 1). The *P2RY4* gene was amplified from 100 ng gDNA using specific primers A and B flanking the open reading frame and 60°C as annealing temperature (Table 1), and sequencing was then carried out on 20 ng of DNA with six primers (A to F) specific for the *P2RY4* gene (Table 1) (3.2 pmol each), 1.5 µl of BigDye™ 5x buffer, 0.8 µl of Ready Reaction Premix, and 10 μl of water. Reactions and readings were performed using an Applied Biosystems 3130 analyzer (Thermo Fisher Scientific). The obtained sequences were compared to the wild-type *P2RY4* gene sequence using SeqScape v2.5 software (Thermo Fisher Scientific) or Ape (A plasmid editor). Minor allele frequency (MAF) values were retrieved from the NCBI dbSNP database.

**TABLE 1:** Sets of specific primers for the human *P2RY4* gene.

Primer name	Sequence (5′ to 3′)	PCR	qRT-PCR	Sequencing
A (forward)	CAG​CTC​TCC​CTA​GTG​CTT​CAA	X		X
B (reverse)	TCT​CCA​GAG​CCT​GGA​AAA​GA	X		X
C (forward)	CAA​GTT​CGT​CCG​CTT​TCT​TT		X	X
D (reverse)	GGC​TAC​GAC​CAA​CCA​AAC​TG		X	X
E (forward)	TGA​ACA​TTG​TCA​ACG​TGG​TCT			X
F (reverse)	CGT​CGA​TAT​TTG​TCC​CCA​GT			X

Primers A and B were used to amplify the entire *P2RY4* coding sequence from all individuals. All primers were used to determine the presence or absence of SNPs in *P2RY4* gene in all individuals. Primers C and D were used the determine the level of P2Y_4_ mRNA in quantitative reverse transcription-PCR experiments in stably transfected 1321N1 cells.

### Functional analysis of the human P2Y_4_ N178T variant

To compare the nucleotide response of N178T variant and wild-type (WT) P2Y_4_ receptors, the *P2RY4* gene sequence was inserted in a pcDNA3 expression vector and transfected in 1321N1 cells, in parallel with WT P2Y_4_ receptor transfection. The transfected 1321N1 cells were harvested in PBS-EDTA (5 mM) at 37°C and then resuspended at 2 × 10^6^ cells/ml in DMEM supplemented with 10% FBS (fetal bovine serum). Two days after transfection, the 1321N1 transfected cells were selected with 400 μg/ml G418 (Life Technologies, Inc., Merelbeke, Belgium) and maintained in the same medium. From the pool of transfected 1321N1 cells, individual clones were isolated by limiting dilution and tested for their P2Y_4_ mRNA expression by quantitative reverse transcription PCR (qRT-PCR). The transfected 1321N1 cells were then loaded with Calcium Sensor Dye 514 (2 μl/ml) and incubated for 30 min at 37°C. UTP 100 µM and ATP 100 µM calcium responses were quantified in N178T P2Y_4_-transfected and WT P2Y_4_-transfected cells and expressed as a percentage of the ionomycin response (5 μg/ml). UTP, ATP, and ionomycin calcium responses were recorded by flow cytometry with a high-speed acquisition during 60 s and quantified for different clonal 1321N1 cell cultures with various expression levels of N178T and WT P2Y_4_ mRNA.

P2Y_4_-GFP constructs were then generated by insertion of the GFP coding sequence in frame with the N178T P2Y_4_ and WT P2Y_4_ coding sequences in a pEGFPN1 vector. To generate these constructs, WT and c.533T>G (p.N178T) mutated P2Y_4_ sequences were produced with a Hind III restriction site inserted directly upstream of the stop codon of the *P2RY4* gene. The obtained constructs were transiently transfected into HEK-293 cells to analyze the expression of GFP-tagged N178T P2Y_4_ and GFP-WT P2Y_4_ receptors, and to compare the GFP signal using Zoe™ Fluorescent Cell Imager (Bio-Rad Laboratories, Temse, Belgium).

### Quantitative reverse transcription PCR

Total mRNAs were extracted from different clonal transfected 1321N1 cell cultures in TRIzol reagent followed by purification with RNeasy kit column (Qiagen, Antwerp, Belgium). mRNA was reverse transcribed using random hexamers and Superscript II Reverse Transcriptase (Invitrogen, Thermo Fisher Scientific, Carlsbad, CA, United States). qRT-PCR amplification mixtures contained 10 ng template cDNA and the specific P2Y_4_ primers C and D (Table 1). Reactions were run on a 7500 Fast Real Time PCR System (Applied Biosystems, Foster City, CA, United States) with an annealing temperature of 60°C for primers C and D. qRT-PCR data were expressed as Ct obtained for P2Y_4_ mRNA in clonal transfected 1321 cells.

### Glucose and insulin tolerance tests

Adiponectin knockout (KO) mice named B6; 129-Adipo^tm1Chan^/J were purchased at JAX, The Jackson Laboratory (Bar Harbor, ME, United States). C57BL/6J P2Y_4_ KO and P2Y_4_/adiponectin double-KO mice were generated in our laboratory. Glucose and insulin tolerance tests were performed with a comparable amount of male and female 9- to 11-week-old P2Y_4_ KO, adiponectin KO, and P2Y_4_/adiponectin double-KO mice. Briefly, 6-h fasted mice were weighed and then injected intraperitoneally with either glucose (2 g/kg body weight) or insulin (0.5 U/kg body weight). Blood samples were collected from the tail vein, and glucose concentrations were measured before and at 10, 20, 30, 60, and 120 min after intraperitoneal injection. Plasma glucose readings were taken from the tail blood using teststrips for OneTouch Ultra2 blood glucose meter (LifeScan, Inc.). The area under the curve was calculated using GraphPad software.

### Statistics

Quantitative parameters are expressed as means ± standard error of the mean (SEM). Between-group differences for quantitative variables were tested using Student’s *t*-test for unpaired series (Welch’s test in case of heteroscedasticity), and the statistical analyses were performed with Prism Software (version 6; GraphPad, CA, United States). All the data obtained from the mouse strain analyses are expressed as means ± SEM. For parallel repeated-measures studies, ANOVA was used with Bonferroni *post hoc* evaluation to determine the significance of individual time points. A two-tailed *p* < 0.05 was considered significant.

### Study approval

The study protocol was approved by the local ethics committee (CCPPRB, Toulouse/Sud-Ouest, file #1-99-48, Feb 2000), with all patients having provided written informed consent prior to participation. The biological sample collection was declared as DC-2008-463 #1 to the French Ministry of Research and to the Regional Health authority. Information and biological samples were collected from individuals according to the principles expressed in the Declaration of Helsinki.

All animal work and *in vivo* models were conducted in accordance with the European Community guidelines for the care and use of laboratory animals and approved by the ethics committee of the Free University of Brussels (current approved protocols 659N and 714N).

## Results

### *P2RY4* gene mutations in individuals from the GENES study

We sequenced the *P2RY4* gene in 50 male patients with CAD and 50 age-matched male control subjects taken from the Génétique et Environnement en Europe du Sud (GENES) study for which different clinical, biological and cardiac parameters at baseline are shown in Table 3. Briefly, CAD patients had a higher systolic blood pressure than controls subjects and were more treated for diabetes, dyslipidemia and hypertension. Among metabolic markers, total cholesterol and LDL-cholesterol were lower in CAD patients, reflecting the effect of the larger proportion of patients treated for dyslipidemia compared to control subjects. However, CAD patients displayed higher levels of triglycerides and lower HDL-C.

Sequencing chromatograms for *P2RY4* are available as Supplementary Material. We identified seven single-nucleotide variants in the *P2RY4* coding sequence (Supplementary Table S1) located in different parts of the corresponding P2Y_4_ amino acid sequence: the N178T substitution is located in the second extracellular loop (e2), S234A and R242R in the third intracellular loop (i3), I247V and R265C in the sixth transmembrane domain (TM6), and W348Stop and P352T in the C-terminus (Figure 1; Table 2).

**TABLE 2:** Identification of *P2RY4* gene variants in control individuals and CAD patients.

Reference	Nucleotide position in CDS and substitution (transcript allele change)	MAF (ToPMeD)	Protein: Amino acid change and position	Protein: Position	Control individuals (*n* = 50): Number of carriers	CAD patients (*n* = 50): Number of carriers
rs1152187	c.533 T>G (A**A**C → A**C**C)	0.331	N178T	e2	16	9
rs3829709	c.700 A>C (**T**CT → **G**CT)	0.133	S234A	i3	6	5
rs3829708	c.726 G>T (CG**C** → CG**A**)	0.100	R242R	i3	6	5
rs56217451	c.739 T>C (**A**TA → **G**TA)	0.040	I247V	TM6	4	2
rs147302991	c.793 G>A (**C**GC → **T**GC)	<0.001	R265C	TM6	1	0
rs41310667	c.1043 C>T (T**G**G → T**A**G)	0.014	W348(Stop)	C-term	5	3
rs72628860	c.1054 G>T (**C**CC → **A**CC)	0.040	P352T	C-term	4	2

Mutations identified after sequencing the human *P2RY4* gene in 50 control individuals and 50 individuals with coronary artery disease (CAD). Identification numbers, minor allele frequencies, and the positions in the human P2Y_4_ receptor (e2, extracellular loop 2; i3, intracellular loop 3; TM6, transmembrane domain 6; C-term, C-terminal region) are indicated for each identified P2Y_4_ variant. CDS (c.), coding DNA sequence; MAF, minor allele frequency.

**FIGURE 2:**
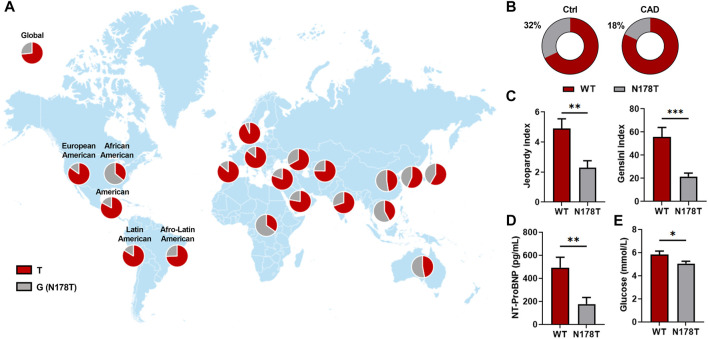
N178T P2Y_4_ variant is associated with reduced cardiac severity scores and lower fasting plasma glucose and NT-proBNP levels in CAD patients. **(A)** Worldwide distribution of c.533T > G (p.N178T) polymorphism in the human *P2RY4* gene corresponding to individuals with the N178T P2Y_4_ receptor mutation, obtained after dbSNP database analysis, compiling data from the ALFA project, 1000Genomes, 8.3KJPN, ExAC, gnomAD, GO Exome Sequencing Project, HapMap, HGDP-CEPH-db, KRGDB, MGP, SGDP_PRJ, and TOPMed. **(B)** On the left, frequency of the N178T mutation in 50 control individuals (Ctrl) and on the right, frequency of the N178T mutation in 50 patients displaying coronary artery disease (CAD). **(C)** Reduced jeopardy and Gensini scores for CAD patients with the N178T mutation in the P2Y_4_ receptor. **(D,E)** Reduced NT-proBNP (pg/ml) and glucose (mmol/L) plasma levels for CAD patients with the N178T mutation in the P2Y_4_ receptor. The data represent the means ± SEM. *p < 0.05; **p < 0.01; ***p < 0.001; Welch’s test.

Among these variants, we decided to focus our analysis on the most common c.533T>G (p.N178T) variant (further referred to as the N178T variant) that was found with a higher frequency in control individuals than in CAD patients (Table 3). The N178T variant frequency was searched for in the dbSNP database to determine its prevalence in certain geographic populations (Figure 2A). The worldwide distribution of the N178T variant revealed that it is very frequent (33.12% in total, 87674/264690, TOPMED) and predominant in Africa and East Asia (Figure 2A). Effectively, the frequency of N178T variant was 65% in Africa and 52.5% in East Asia in the dbSNP database. In our study population, representative of the south-west France, we observed a reduction of N178T variant frequency in CAD patients compared with control individuals. The frequency was 32% in the 50 control individuals versus 18% in the 50 CAD patients (*p* = 0.11; Figure 2B; Table 3).

**TABLE 3:** Baseline characteristics of the population from the GENES case-control study, according to status for CAD.

	Control (No CAD)*n* = 50	Case (CAD) *n* = 50	*p*-value^a^
Clinical			
Age, years	60.6 (1.2)	61.0 (1.0)	0.78
Body mass index, kg/m^2^	26.8 (0.66)	27.9 (0.59)	0.24
Waist circumference, cm	96.0 (1.6)	99.7 (1.5)	0.11
Biological (fasting)			
Glucose, mmol/L	5.37 (0.11)	5.70 (0.25)	0.24^b^
Triglycerides, g/L	1.34 (0.20)	1.70 (0.12)	0.0015^c^**
Total cholesterol, g/L	2.23 (0.06)	2.01 (0.06)	0.016*
LDL-C, g/L	1.43 (0.05)	1.25 (0.06)	0.018*
HDL-C, g/L	0.55 (0.02)	0.43 (0.01)	0.001**
Cardiac parameters			
Resting heart rate, bpm	63.0 (1.2)	63.0 (1.5)	0.99
Systolic blood pressure, mmhg	134 (2.5)	144 (3.4)	0.016*
NT-proBNP, pg/mL	n.a	436.5 (75.5)	—
hs-TnT, pg/mL	n.a	81.1 (30.1)	—
Treatments			
Diabetes, n (%)	2 (4%)	13 (26%)	0.0021**
Dyslipidemia, n (%)	9 (18%)	34 (68%)	0.001**
Hypertension, n (%)	16 (32%)	35 (70%)	0.001**
N178T, n (%)	16 (32%)	9 (18%)	0.11

Variables were measured at baseline (i.e., when individuals were first included in the GENES cohort). For continuous variables, values are expressed as mean ± SEM. For categorical variables, values are expressed as number with frequency (%) in parentheses.

^a^ Paired Student’s *t*-test, unless.

^b^ Welch’s test in case of heteroscedasticity.

c Analyses performed on log transformed data. *p < 0.05; **p < 0.01.

Bpm, beats per minute; CAD, coronary artery disease; HDL-C, high-density lipoprotein cholesterol; hs-TnT, high-sensitivity cardiac troponin T; LDL-C, low-density lipoprotein cholesterol; n.a., not available; NT-proBNP, N-terminal pro-brain natriuretic peptide.

### Cardiometabolic health in coronary patients carrying the N178T variant of P2Y_4_ receptor

In the CAD patients of our study population, carriers of the N178T variant had less severe coronary artery atherosclerosis compared to WT patients, as estimated by the jeopardy score documenting the diffusion of coronary heart disease lesions, and the Gensini score documenting the number, location, and degree of stenosis of coronary artery lesions (Figure 2C). Regarding markers of cardiac function, the resting heart rates were lower in CAD patients carrying the N178T variant (Table 4, 55.5 ± 3.4 versus 64.6 ± 1.5 bpm in WT; mean ± SEM, *p* = 0.015) as well as the plasma level of NT-proBNP (Figure 2D; Table 4, 177.6 ± 56.3 versus 493.3 ± 89.1 pg/ml in WT; mean ± SEM, *p* = 0.004). Regarding plasma biological parameters, the presence of the N178T variant was associated with a significant lower concentration of fasting plasma glucose (Figure 2E; Table 4). No differences were seen between N178T and WT patients regarding plasma lipids levels or anthropometric parameters such as age, waist circumference, and body mass index (Table 4).

**TABLE 4:** Baseline characteristics of CAD patients, according to N178T status.

	CAD WT *n* = 41	CAD N178T *n* = 9	*p*-value^a^
Clinical			
Age, years	61.7 (1.0)	57.7 (3.4)	0.12
Body mass index, kg/m^2^	27.7 (0.7)	28.7 (1.3)	0.51
Waist circumference, cm	99.6 (1.7)	100.0 (4.0)	0.93
Biological (fasting)			
Glucose, mmol/L	5.84 (0.30)	5.05 (0.20)	0.034^b^*
Triglycerides, g/L	1.68 (0.13)	1.74 (0.29)	0.79^c^
Total cholesterol, g/L	1.99 (0.07)	2.13 (0.13)	0.41
LDL-C, g/L	1.22 (0.07)	1.34 (0.11)	0.45
HDL-C, g/L	0.43 (0.02)	0.44 (0.02)	0.79
Cardiac parameters			
Resting heart rate, bpm	64.6 (1.5)	55.5 (3.4)	0.015*
Systolic blood pressure, mmhg	145 (3.6)	138 (9.3)	0.41
NT-proBNP, pg/mL	493.3 (89.1)	177.6 (56.3)	0.004^b^**
hs-TnT, pg/mL	92.6 (36.5)	28.9 (10.2)	0.10^b^
Treatments			
Diabetes, n (%)	12 (29%)	1 (11%)	0.26
Dyslipidemia, n (%)	27 (66%)	7 (78%)	0.48
Hypertension, n (%)	30 (73%)	5 (56%)	0.29

Variables were measured at baseline (i.e., when individuals were first included in the GENES cohort). For continuous variables, values are expressed as mean ± SEM. For categorical variables, values are expressed as number with frequency (%) in parentheses.

^a^ Paired Student’s *t*-test, unless.

^b^ Welch’s test in case of heteroscedasticity.

^c^ Analyses performed on log transformed data. *p < 0.05; **p < 0.01.

Bpm, beats per minute; CAD, coronary artery disease; HDL-C, high-density lipoprotein cholesterol; hs-TnT, high-sensitivity cardiac troponin T; LDL-C, low-density lipoprotein cholesterol; NT-proBNP, N-terminal pro-brain natriuretic peptide.

### N178T polymorphism is a loss-of-function mutation of the P2Y_4_ receptor

**FIGURE 3:**
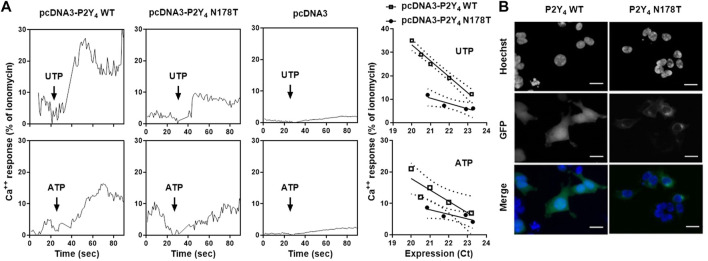
N178T polymorphism is a loss-of-function mutation for the human P2Y_4_ receptor. **(A)** Reduced UTP and ATP responses in 1321N1 cells expressing the N178T P2Y_4_ mutant. The calcium responses to 100 µM UTP or 100 µM ATP were compared in 1321N1 cells expressing the WT P2Y_4_ receptor or the N178T P2Y_4_ receptor. Left graphs, UTP and ATP calcium response in a pool of 1321N1 cells transfected with pcDNA3-WT P2Y_4_ or pcDNA3-N178T P2Y_4_, selected with G418 and displaying a comparable Ct value for P2Y_4_ mRNA expression in qRT-PCR experiments. UTP and ATP calcium response was also tested in a pool of 1321N1 cells transfected with the pcDNA3 vector alone. Right graphs, UTP, and ATP calcium responses in individual 1321N1 transfected clones expressing various levels (expressed in Ct values from qRT-PCR experiments) of WT or N178T P2Y_4_ receptor. **(B)** Reduced fluorescent signal of GFP-tagged N178T P2Y_4_ receptors compared to GFP-tagged WT P2Y_4_ receptors in transfected HEK-293 cells. Images were obtained using Zoe™ Fluorescent Cell Imager (scale bar = 25 µm).

We have previously demonstrated that knockout of the P2Y_4_ receptor in mice induced cardioprotection in the LAD ligation model (Horckmans et al., 2015). A possible link could exist between reduced cardiac severity scores associated with the N178T P2Y_4_ human receptor and the cardioprotective effect of P2Y_4_ receptor loss in mouse. We hence compared the nucleotide response of the N178T and WT P2Y_4_ human receptors. The N178T P2Y_4_ coding sequence was inserted in a pcDNA3 expression vector and transfected into 1321N1 astrocytoma cells, in parallel with WT P2Y_4_ receptor-pcDNA3 transfection of 1321N1 cells. 1321N1 astrocytoma cells are commonly used for nucleotide receptor transfection due to their unique lack of endogenous nucleotide receptors. We observed a strong reduction of UTP and ATP calcium responses in a pool of pcDNA3-N178T P2Y_4_ compared to pcDNA3-WT P2Y_4_ G418-resistant transfected cells (Figure 3A). These reduced calcium responses to UTP and ATP were observed in individual G418-resistant clones of transfected 1321N1 cells that had various expression levels (expressed as the Ct obtained in qPCR experiments) (Figure 3A, right panels). We also analyzed the expression of green fluorescent protein (GFP)-N178T P2Y_4_ and GFP-WT P2Y_4_ receptors after construction and transient transfection of their respective pEGFPN1 expression vectors in HEK-293 cells. Microscopy analysis using Zoe™ Fluorescent Cell Imager revealed a loss of fluorescent signal for the N178T P2Y_4_ receptor compared to the WT P2Y_4_ receptor (Figure 3B).

### Lack of P2Y_4_ correlates with improved glucose tolerance and insulin sensitivity in mice

**FIGURE 4:**
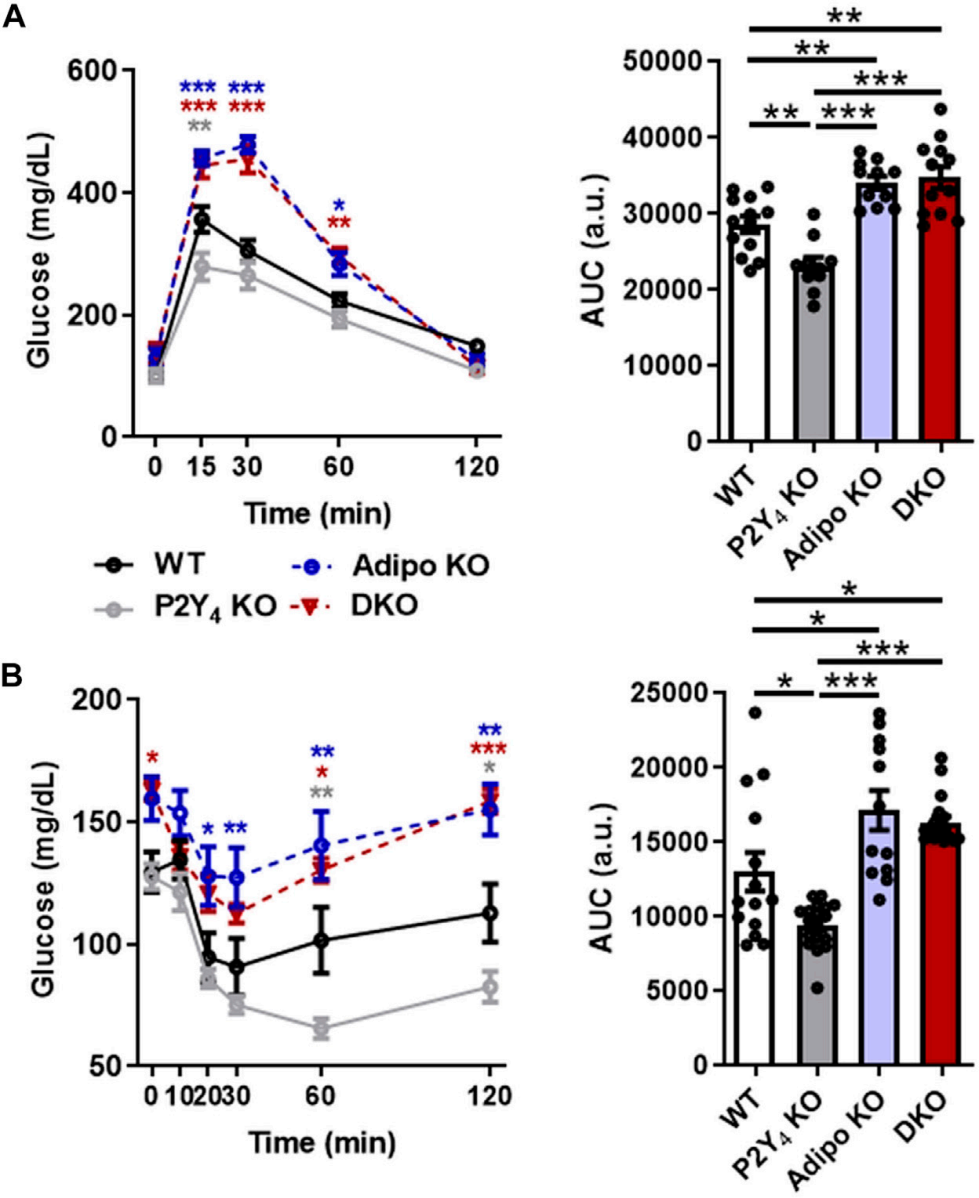
Loss of mouse P2Y_4_ receptor improves glucose metabolism. **(A)** Glucose tolerance test in P2Y_4_ KO mice. After 6 h-fasting, glucose (2 g/kg) was intraperitoneally injected, and blood glucose levels were monitored in WT, P2Y_4_ KO, adiponectin KO (AdipoKO) and P2Y_4_/adiponectin double-KO (DKO) mice (*n* = 12–14). Statistical differences from the control values (WT mice) are shown. **(B)** Insulin tolerance test in P2Y_4_ KO mice. After 6 h-fasting, insulin (0.5 U/kg) was intraperitoneally injected, and blood glucose levels were monitored (*n* = 8–14). The area under the curve was quantified both in panels **(A,B)** with GraphPad Prism software (a.u., arbitrary unit). The data represent the means ± SEM. *p < 0.05; **p < 0.01; ***p < 0.001. Values for individual time points (left graph) and area under the curve (right graph) were analyzed using respectively one-way ANOVA test and two-way ANOVA test with Bonferroni *post hoc* evaluation.

A possible association between cardioprotection and glucose homeostasis was further investigated in P2Y_4_ KO mice using glucose and insulin tolerance tests (Figures 4A,B). P2Y_4_ KO mice, characterized by adiponectin overexpression (Lemaire et al., 2017), had a significantly increased glucose tolerance and insulin sensitivity compared to their WT littermate controls (Figures 4A,B). Conversely, adiponectin KO and adiponectin/P2Y_4_ double-KO (DKO) mice exhibited similar impaired glucose tolerance and insulin sensitivity, compared with P2Y_4_ KO mice (Figures 4A,B). In glucose tolerance tests, the difference between WT and P2Y_4_ KO mice is only significant 15 min after glucose injection (Figure 4A, left panel), and on the area under the curve (Figure 4A, right panel). Significant interindividual variability was observed in insulin tolerance tests, especially for WT and adiponectin KO mice (Figure 4B). We observed a higher insulin sensitivity in P2Y_4_ KO mice, significant 60 and 120 min after insulin injection, and impaired insulin sensitivity in adiponectin KO and DKO mice, compared to WT mice (Figure 4B).

## Discussion

Whereas nucleotide receptors such as the P2Y_1_ and P2Y_2_ receptors are ubiquitously expressed, the P2Y_4_ subtype has a more confined tissue distribution restricted mainly to the intestine, lung, heart, cardiac adipose tissue, pancreas and placenta (Communi et al., 1995; Suarez-Huerta et al., 2001; Horckmans et al., 2012a; Lemaire et al., 2017). The human P2Y_4_ receptor is a UTP receptor, while the mouse P2Y_4_ receptor is activated by both ATP and UTP (Suarez-Huerta et al., 2001). We have extensively studied the cardiac phenotype of P2Y_4_ KO mice, which are characterized by reduced post-natal heart development (Horckmans et al., 2012a), decreased resistance to exercise (Horckmans et al., 2012b), and protection against myocardial infarction (Horckmans et al., 2015). More specifically, P2Y_4_ KO mice exhibit smaller infarcts in the LAD ligation model, as well as a significant decrease in cardiac inflammation and adiponectin overexpression (Horckmans et al., 2015).

Among the mutations identified in the human *P2RY4* gene in the present study, we focused our attention on the single nucleotide polymorphism c.533T>G (p.N178T) (rs1152187), corresponding to N178T substitution in the second extracellular loop of the human P2Y_4_ receptor. This particular polymorphism was detected significantly less in CAD patients than in control individuals, albeit in a limited sequencing of the human *P2RY4* gene in 50 normal individuals and 50 patients. This missense P2Y_4_ variant is widely present worldwide in the general population, and it is predominant in Africa and East Asia.

The frequency of N178T polymorphism in control individuals reported in the present study population, representative of the south-west France, is comparable to its mean worldwide distribution, with a frequency of 33%. Although ischemic heart disease remains relatively uncommon in sub-Saharan Africa, its incidence is rising due to the increasing prevalence of risk factors (Onen, 2013; Yao et al., 2022). The epidemiology of ischemic heart disease in Africa remains largely enigmatic due to a lack of cardiologists and reliable health statistics (Keates et al., 2017). Higher stroke rates but lower coronary heart disease have been observed in Asian countries compared to Western countries (Ueshima et al., 2018). Analysis of outcomes after acute myocardial infarction (AMI) between South Asian, Chinese, and white patients has revealed significantly lower long-term mortality in South Asian patients (Khan et al., 2010). Naturally, the genetics of CAD is very complex, and heart disease statistics depend on multiple risk factors. No direct link can thus be made between these AMI incidence data and the worldwide distribution of the highly common N178T P2Y_4_ receptor polymorphism.

Most interestingly, the presence of the N178T P2Y_4_ variant in CAD patients is correlated with reduced jeopardy and Gensini scores of CAD severity. We investigated whether the N178T substitution could affect human P2Y_4_ receptor activity, given that mice deficient in the P2Y_4_ receptor are protected from myocardial infarction (Horckmans et al., 2015). We performed functional experiments on 1321N1 cell lines stably expressing both the wild-type and the N178T human P2Y_4_ receptor. A loss of function of the N178T P2Y_4_ receptor was observed in response to both UTP and ATP. ATP binds both human and rat P2Y_4_ receptor homologs, but whereas ATP activates the rat and mouse receptors, it antagonizes the human receptor (Herold et al., 2004). Interestingly, the N178T polymorphism is located in the second extracellular loop of the human P2Y_4_ receptor, which has been reported to be a major determinant of agonist versus antagonist activity of ATP in rat and human P2Y_4_ homologs (Herold et al., 2004). When a chimeric receptor is generated in which the second extracellular loop of the human P2Y_4_ receptor is replaced with the corresponding region of the rat P2Y_4_ receptor, ATP is fully agonistic toward the generated chimera (Herold et al., 2004). ATP is described as a partial agonist and even an antagonist of the human wild-type P2Y_4_ receptor depending on its membrane expression level (Communi et al., 1995; Herold et al., 2004). The loss-of-function N178T polymorphism of the human P2Y_4_ receptor identified in the present study led to its decreased membrane expression, but this mutation could also affect the structure of the second extracellular loop, which is important for nucleotide affinity.

Although reduced jeopardy and Gensini scores were observed in CAD patients with the N178T P2Y_4_ variant, comparison with protection against myocardial infarction in P2Y_4_ KO ischemic mice must be undertaken with caution. Among the other cardiac and metabolic parameters analyzed in CAD patients, we observed slower heart rates and reduced glucose plasma levels in patients with the N178T P2Y_4_ receptor. Interestingly, glucose and insulin tolerance tests demonstrated that P2Y_4_ KO mice, characterized by adiponectin overexpression, had lower glycemia and a higher sensitivity to insulin than WT mice. As expected, insulin resistance was identified in adiponectin KO mice and higher sensitivity to insulin resulting from P2Y_4_ loss was no longer observed in the absence of adiponectin in adiponectin/P2Y_4_ double-KO mice.

The present study shows that the P2Y_4_ receptor could be considered as a member of the P2Y receptor family involved in the regulation of glucose metabolism. The P2Y_4_ receptor is able to couple to G_q/11_ proteins, but also to G_i_ proteins (Communi et al., 1996), which are known to have an antilipolytic effect. Another study has demonstrated that mice lacking adipocyte P2Y_6_ receptor were protected from diet-induced obesity and were characterized by improved glucose tolerance and insulin sensitivity (Jain et al., 2020). It has been also shown that P2Y_2_ and P2Y_4_ receptors can regulate Cl^−^ and K^+^ channels and intracellular Ca^2+^ signalling in pancreatic ducts (Hede et al., 2005; Novak, 2008). More generally, the activation of nucleotide receptors by ATP participates in the potentiation of glucose-stimulated insulin secretion by increasing the exocytosis of insulin granules in pancreatic *β*-Cells (Mesto et al., 2022).

Among the measured parameters that were significantly different between wild-type and N178T CAD patients, NT-proBNP was particularly reduced in N178T CAD patients. The level of NT-proBNP is considered to be a cardiovascular risk factor and of prognostic value in patients with previous myocardial infarction (Reinstadler et al., 2016; Cao et al., 2022). The identification of biomarkers related to a high-risk population and prognosis prediction is a major factor in CAD preventive measures. NT-proBNP is a neurohormone synthesized and released by the heart in response to increased wall tension (Rorth et al., 2020). NT-proBNP is thus a key marker for heart failure but also a risk marker for the prediction of major adverse cardiovascular events (Redfors et al., 2018; Liu et al., 2021). Ischemic myocardium secretes elevated levels of NT-proBNP, even in the absence of left ventricular dysfunction (Goetze et al., 2003). The risk of a recurrent cardiac event is significantly higher in patients with elevated NT-proBNP levels (Cao et al., 2022). The level of specific biomarkers during a chronic or later phase is a better predictor of prognosis of cardiac function than their level during the acute phase. Our study could contribute to the NT-proBNP level being taken into account in the risk assessment of patients with previous MI, in association with the detection of *P2RY4* gene polymorphisms.

The identification of this specific N178T P2Y_4_ variant could be useful and contributive when combined with other known cardiac gene polymorphisms to predict the severity of infarction in humans. A personal approach based on individual genetic factors and metabolic parameters is important in the treatment of myocardial infarction. Nucleotide receptors can be considered to be key players in the regulation of cardioprotection and glucose homeostasis. Nucleotide receptors such as the P2Y_4_ subtype, expressed in adipose tissues and regulating the production of adipokines, can facilitate the onset of insulin resistance. Antagonists of P2Y receptors could thus have therapeutic applications in the treatment of type 2 diabetes. The present identification of a cardioprotective loss-of-function polymorphism of the human *P2RY4* gene correlated with reduced plasma levels of fasting glucose and NT-proBNP in CAD patients, as well as the insulin sensitivity in P2Y_4_-deficient mice, support the notion that this nucleotide receptor constitutes another candidate for the development of such antagonists.

## Data Availability

The original contributions presented in the study are included in the article/Supplementary Materials, further inquiries can be directed to the corresponding author.
