# Supplementary material for: Loss-of-function N178T variant of the human P2Y4 receptor is associated with decreased severity of coronary artery disease and improved glucose homeostasis
Source: Front Pharmacol. 2022 Dec 2;13:1049696. doi: 10.3389/fphar.2022.1049696 (PMC9755162; doi:10.3389/fphar.2022.1049696)
Supplement: Supplementary file 2 [file Table1.DOCX]

**Supplemental Table 1**

**Identified polymorphisms in *P2RY4* gene in control and case (CAD) individuals (ID)**

| **Control ID** | **N178T** c.533 (T > G) AAC → ACC | **S234A** c.700 (A → C) TCT → GCT | **R242R** c.726 (G >T) CGC (Arg) → CGA | **I247V** c.739 (T > C) ATA → GTA | **R265C** c.793 (C > T) CGC →TGC | **W348(stop)** c.1043 (G > A) TGG → TAG | **P352T** c.1054 (C > A) CCC → ACC | **primers used for sequencing** |
| --- | --- | --- | --- | --- | --- | --- | --- | --- |
| 9 | 0 | 0 | 0 | 0 | 0 | 0 | 0 | C,D |
| 42 | 1 | 0 | 0 | 1 | 0 | 0 | 1 | B,C,D |
| 63 | 0 | 0 | 0 | 0 | 0 | 0 | 0 | B,C,D |
| 99 | 1 | 0 | 0 | 1 | 0 | 0 | 1 | A,C,D |
| 127 | 1 | 1 | 1 | 0 | 0 | 1 | 0 | A,B,C,F |
| 143 | 0 | 0 | 0 | 0 | 0 | 0 | 0 | A,B,E |
| 148 | 0 | 0 | 0 | 0 | 0 | 0 | 0 | C,D |
| 182 | 1 | 0 | 0 | 0 | 0 | 0 | 0 | A,C,D |
| 224 | 1 | 1 | 1 | 0 | 0 | 1 | 0 | A,B,C,D |
| 243 | 0 | 0 | 0 | 0 | 0 | 0 | 0 | A,B,C,D |
| 244 | 1 | 0 | 0 | 1 | 0 | 0 | 1 | C,D |
| 267 | 1 | 0 | 0 | 0 | 0 | 0 | 0 | A,C,D |
| 281 | 0 | 0 | 0 | 0 | 0 | 0 | 0 | A,C,D |
| 306 | 0 | 0 | 0 | 0 | 0 | 0 | 0 | C,D |
| 317 | 0 | 0 | 0 | 0 | 0 | 0 | 0 | C,D |
| 326 | 1 | 1 | 1 | 0 | 0 | 0 | 0 | C,D |
| 343 | 0 | 0 | 0 | 0 | 0 | 0 | 0 | C,D |
| 348 | 0 | 0 | 0 | 0 | 0 | 0 | 0 | B,C,D |
| 382 | 1 | 1 | 1 | 0 | 0 | 1 | 0 | A,B |
| 411 | 1 | 1 | 1 | 0 | 0 | 1 | 0 | C,D |
| 431 | 0 | 0 | 0 | 0 | 0 | 0 | 0 | C,D |
| 468 | 0 | 0 | 0 | 0 | 0 | 0 | 0 | C,D |
| 482 | 1 | 0 | 0 | 0 | 0 | 0 | 0 | B,C,D |
| 499 | 0 | 0 | 0 | 0 | 0 | 0 | 0 | C,D |
| 524 | 0 | 0 | 0 | 0 | 0 | 0 | 0 | C,D |
| 540 | 0 | 0 | 0 | 0 | 0 | 0 | 0 | C,D |
| 547 | 0 | 0 | 0 | 0 | 0 | 0 | 0 | C,D |
| 562 | 0 | 0 | 0 | 0 | 0 | 0 | 0 | A,B |
| 571 | 1 | 0 | 0 | 0 | 0 | 0 | 0 | C,D |
| 572 | 0 | 0 | 0 | 0 | 1 | 0 | 0 | C,D |
| 579 | 0 | 0 | 0 | 0 | 0 | 0 | 0 | C,D |
| 587 | 1 | 0 | 0 | 0 | 0 | 0 | 0 | C,D |
| 593 | 0 | 0 | 0 | 0 | 0 | 0 | 0 | C,D |
| 596 | 0 | 0 | 0 | 0 | 0 | 0 | 0 | C,D |
| 603 | 0 | 0 | 0 | 0 | 0 | 0 | 0 | A,B |
| 641 | 0 | 0 | 0 | 0 | 0 | 0 | 0 | C,D |
| 673 | 0 | 0 | 0 | 0 | 0 | 0 | 0 | C,D |
| 674 | 1 | 0 | 0 | 1 | 0 | 0 | 1 | C,D |
| 715 | 0 | 0 | 0 | 0 | 0 | 0 | 0 | C,D |
| 739 | 0 | 0 | 0 | 0 | 0 | 0 | 0 | C,D |
| 757 | 0 | 0 | 0 | 0 | 0 | 0 | 0 | A,C |
| 767 | 0 | 0 | 0 | 0 | 0 | 0 | 0 | C,D |
| 769 | 0 | 0 | 0 | 0 | 0 | 0 | 0 | A,B |
| 772 | 0 | 0 | 0 | 0 | 0 | 0 | 0 | A,C |
| 773 | 1 | 1 | 1 | 0 | 0 | 1 | 0 | A,B,C |
| 778 | 0 | 0 | 0 | 0 | 0 | 0 | 0 | C,D |
| 792 | 0 | 0 | 0 | 0 | 0 | 0 | 0 | C,D |
| 804 | 0 | 0 | 0 | 0 | 0 | 0 | 0 | C,D |
| 807 | 0 | 0 | 0 | 0 | 0 | 0 | 0 | A,B |
| 816 | 1 | 0 | 0 | 0 | 0 | 0 | 0 | A,B |
| **SNP Number** | 16 | 6 | 6 | 4 | 1 | 5 | 4 |  |

| **CASE (CAD) ID** | **N178T** c.533 (T > G) AAC → ACC | **S234A** c.700 (A → C) TCT → GCT | **R242R** c.726 (G >T) CGC (Arg) → CGA | **I247V** c.739 (T > C) ATA → GTA | **R265C** c.793 (C > T) CGC →TGC | **W348(stop)** c.1043 (G > A) TGG → TAG | **P352T** c.1054 (C > A) CCC → ACC | **primers used for sequencing** |
| --- | --- | --- | --- | --- | --- | --- | --- | --- |
| 1_37026 | 0 | 0 | 0 | 0 | 0 | 0 | 0 | A,B,C,D |
| 1_57009 | 0 | 0 | 0 | 0 | 0 | 0 | 0 | C,D |
| 1_58416 | 1 | 1 | 1 | 0 | 0 | 0 | 0 | A,B,C,D |
| 1_59969 | 0 | 0 | 0 | 0 | 0 | 0 | 0 | A,B,C,D,E |
| 1_60553 | 0 | 0 | 0 | 0 | 0 | 0 | 0 | A,B,C,D |
| 1_63593 | 1 | 1 | 1 | 0 | 0 | 1 | 0 | A,B,C,D,E,F |
| 1_64520 | 0 | 0 | 0 | 0 | 0 | 0 | 0 | B,D |
| 1_68241 | 0 | 0 | 0 | 0 | 0 | 0 | 0 | A,C,D,E |
| 1_68839 | 0 | 0 | 0 | 0 | 0 | 0 | 0 | A,B,D,E |
| 1_70137 | 0 | 0 | 0 | 0 | 0 | 0 | 0 | C,D |
| 2_09018 | 0 | 0 | 0 | 0 | 0 | 0 | 0 | C,D |
| 2_13737 | 0 | 0 | 0 | 0 | 0 | 0 | 0 | C,D |
| 2_15069 | 1 | 1 | 1 | 0 | 0 | 0 | 0 | C,D |
| 2_17178 | 0 | 0 | 0 | 0 | 0 | 0 | 0 | C,D |
| 2_20863 | 0 | 0 | 0 | 0 | 0 | 0 | 0 | C,D |
| 2_28469 | 0 | 0 | 0 | 0 | 0 | 0 | 0 | C,D |
| 2_34847 | 1 | 1 | 1 | 0 | 0 | 1 | 0 | C,D |
| 2_40892 | 0 | 0 | 0 | 0 | 0 | 0 | 0 | C,D |
| 2_41536 | 0 | 0 | 0 | 0 | 0 | 0 | 0 | C,D |
| 2_42458 | 0 | 0 | 0 | 0 | 0 | 0 | 0 | C,D |
| 2_45965 | 0 | 0 | 0 | 0 | 0 | 0 | 0 | C,D |
| 2_51944 | 0 | 0 | 0 | 0 | 0 | 0 | 0 | C,D |
| 2_55412 | 1 | 0 | 0 | 1 | 0 | 0 | 1 | C,D |
| 2_60238 | 0 | 0 | 0 | 0 | 0 | 0 | 0 | C,D |
| 2_66691 | 0 | 0 | 0 | 0 | 0 | 0 | 0 | A,C |
| 2_68753 | 0 | 0 | 0 | 0 | 0 | 0 | 0 | C,D |
| 2_77411 | 0 | 0 | 0 | 0 | 0 | 0 | 0 | C,D |
| 2_77436 | 0 | 0 | 0 | 0 | 0 | 0 | 0 | C,D |
| 2_78163 | 0 | 0 | 0 | 0 | 0 | 0 | 0 | C,D |
| 3_01295 | 0 | 0 | 0 | 0 | 0 | 0 | 0 | C,D |
| 3_02279 | 0 | 0 | 0 | 0 | 0 | 0 | 0 | A,B |
| 3_03006 | 0 | 0 | 0 | 0 | 0 | 0 | 0 | C,D |
| 3_10727 | 0 | 0 | 0 | 0 | 0 | 0 | 0 | A,B |
| 3_10728 | 0 | 0 | 0 | 0 | 0 | 0 | 0 | C,D |
| 3_11886 | 0 | 0 | 0 | 0 | 0 | 0 | 0 | C,D |
| 3_12518 | 0 | 0 | 0 | 0 | 0 | 0 | 0 | A,C,D |
| 3_13208 | 0 | 0 | 0 | 0 | 0 | 0 | 0 | C,D |
| 3_18096 | 0 | 0 | 0 | 0 | 0 | 0 | 0 | C,D,F |
| 3_18097 | 0 | 0 | 0 | 0 | 0 | 0 | 0 | A,B |
| 3_24706 | 0 | 0 | 0 | 0 | 0 | 0 | 0 | A,C |
| 3_33973 | 0 | 0 | 0 | 0 | 0 | 0 | 0 | C,D |
| 3_36755 | 0 | 0 | 0 | 0 | 0 | 0 | 0 | C,D |
| 3_36757 | 0 | 0 | 0 | 0 | 0 | 0 | 0 | C,D |
| 3_40881 | 0 | 0 | 0 | 0 | 0 | 0 | 0 | C,D |
| 3_40925 | 1 | 0 | 0 | 1 | 0 | 0 | 1 | C,D |
| 3_55158 | 0 | 0 | 0 | 0 | 0 | 0 | 0 | C,D |
| 3_56719 | 1 | 1 | 1 | 0 | 0 | 1 | 0 | A,B |
| 3_58277 | 1 | 0 | 0 | 0 | 0 | 0 | 0 | C,D |
| 3_58650 | 1 | 0 | 0 | 0 | 0 | 0 | 0 | C,D |
| 3_60477 | 0 | 0 | 0 | 0 | 0 | 0 | 0 | A,B |
| **SNP Number** | **9** | **5** | **5** | **2** | **0** | **3** | **2** |  |
